# Therapeutic Role of Functional Massage in Attenuating Exercise-Induced Neuromuscular Fatigue

**DOI:** 10.3390/bioengineering12080880

**Published:** 2025-08-16

**Authors:** Zahraa Darwich, Alaa Issa, Emma Parkin, Jada Young, Marie Eve Pepin, Moh H. Malek

**Affiliations:** 1 Physical Therapy Program, Department of Health Care Sciences, Wayne State University, College of Pharmacy and Health Sciences, Detroit, MI 48201, USA; 2 Integrative Physiology of Exercise Laboratory, Department of Health Care Sciences, Wayne State University, College of Pharmacy and Health Sciences, Detroit, MI 48201, USA

**Keywords:** physical activity science, functional physiology, movement science, sports physiology

## Abstract

Background: Functional massage is a soft tissue intervention that combines tissue compression with specific joint movements to enhance muscle function, improve joint mobility and reduce pain. The physical working capacity at the fatigue threshold (PWC_FT_) uses surface electromyography to determine the highest exercise intensity that can be sustained indefinitely. The purpose of this study, therefore, was to examine the influence of FM on a multi-joint exercise such as cycle ergometry. Methods: Twelve healthy college-aged men volunteered for the current study. On two occasions, separated by seven days and in randomized order, subjects either completed a 14 min FM on both legs prior to an incremental cycle ergometer test to determine PWC_FT_, or rested for 14 min before performing the same cycling test. Results: The paired samples *t*-tests revealed a significant (*p <* 0.05) difference for the absolute and relative PWC_FT_ values between the no-FM and FM conditions. Conclusions: These results indicate that FM may delay the onset of neuromuscular fatigue for whole-body exercise.

## 1. Introduction

Functional massage (FM) is a soft tissue intervention that combines tissue compression with specific joint movements to enhance muscle function, improve joint mobility and reduce pain [1,2,3,4]. In addition to the therapeutic effects of the massage applied to the muscle(s) and surrounding soft tissues, the technique also incorporates limb movement to facilitate either contraction, or a stretch of the targeted muscle, further enhancing its functional benefit. [1,2,3,4]. During the inflammatory stage, shortening the muscle during the massage can help mitigate the adverse effects of immobility without disrupting the healing process. In contrast, during the remodeling phase, lengthening the tissue is often advised to support collagen realignment, which may improve the tissue’s structural integrity and extensibility [1,2,3,4]. By reducing adhesions and soft tissue restrictions, FM may enhance muscle activation and overall neuromuscular performance. Additionally, FM may reduce excessive protective neural activity, such as involuntary muscle guarding, while promoting efficient voluntary neuromuscular activation. This dual effect can optimize both relaxation and functional performance [1,2,3,4]. Finally, incorporating muscle contractions during the hands-off portion of the technique can offer additional functional benefits such as promoting neuromuscular reeducation, improving local circulation and fluid dynamics, and enhancing the delivery of nutrients to healing tissues. Despite its widespread use in rehabilitative and physical therapy clinics, evidence-based data to support the use of FM are limited.

Surface electromyography (EMG) is a non-invasive methodology to assess motor unit activation and recruitment during isometric, isokinetic, or dynamic muscle action [5]. The domains of EMG signal are its amplitude and frequency [5]. For continuous exercise such as treadmill running or cycle ergometry studies have shown that the EMG amplitude is highly reliable, whereas the EMG mean power frequency has low reliability [5]. Typically, the patterns of responses for the EMG amplitude versus exercise intensity (i.e., power output or isometric force) is characterized by using polynomial regression [6,7]. Alternatively, deVries and colleagues [8,9,10] proposed the physical working capacity at the fatigue threshold (PWC_FT_), which plots the EMG amplitude versus time for each power output during an incremental cycle ergometry test. Thereafter, linear regression analysis is performed for each power output and the highest power output with a non-significant slope is identified [8,9,10]. Then, the lower power output with a significant slope is identified [8,9,10]. The PWC_FT_ is the average of these two power outputs. Theoretically, the PWC_FT_ is the highest power output that a subject can maintain indefinitely without an increase in the EMG amplitude [8,9,10]. Indeed, Briscoe and colleagues validated the PWC_FT_, in a group of healthy college-aged men and women, by reporting an increase in the EMG amplitude versus time relationship for an exercise intensity above the PWC_FT_ [11].

The PWC_FT_ is an index that demarcates between moderate and heavy exercise domains [12]. Indeed, Miller et al. [13] reported that the onset of the PWC_FT_ was ~14% higher than the onset of the gas exchange threshold (i.e., noninvasive method of determining lactate threshold). In addition, the authors found a strong positive correlation (r = 0.74) between the PWC_FT_ and gas exchange threshold. These findings support the use of PWC_FT_ as a reliable, noninvasive indicator of neuromuscular fatigue threshold and a valuable alternative to traditional metabolic markers such as oxygen uptake or blood lactate for assessing endurance performance. It should be noted, however, that over the years investigators have developed analogs to the PWC_FT_, which use oxygen uptake [13,14], heart rate [15], pain [16], and rating of perceived exertion [14] as the primary fatigue indices. Taken together, the PWC_FT_ provides a versatile method of determining fatigue with various indices.

Initially, deVries et al. [8,9,10] used a 2 min stage; however, Evetovich and colleagues [17] wanted to determine whether extending the duration to 3 or 4 min would result in different PWC_FT_ values. The authors reported that the PWC_FT_ values from the three different durations were significantly different from one another. Indeed, for the 2 min duration, the PWC_FT_ was ~9% to ~23% higher than the 3 min and 4 min durations, respectively [17]. To further evaluate the PWC_FT_, a subset of the subjects performed continuous cycle ergometry up to 60 min at the PWC_FT_ determined from the 2 and 4 min incremental protocols. Evetovich et al. [17] reported that subjects were able to ride, on average, for 13 min for the PWC_FT_ determined from the 2 min protocol, whereas subjects were able to ride, on average, for 32 min for the PWC_FT_ determined from the 4 min protocol. The authors concluded that the duration of incremental test to determine PWC_FT_ needs to be considered as a potential variable in future studies [17]. Over 90% of studies examining the PWC_FT_ have used cycle ergometry, a mode of exercise that primarily activates the quadriceps femoris muscles to generate force. Thus, it is important to determine whether these muscles exhibit distinct PWC_FT_ values as these potential differences could have implications for interpreting fatigue during cycling. Indeed, Housh and colleagues [18] calculated a PWC_FT_ value for the three superficial quadriceps femoris muscles during incremental cycle ergometry. The authors reported no significant mean differences in the PWC_FT_ values between the three muscles [18]. Housh et al. [18] concluded that quadricep femoris muscles respond as a unit with respect to the onset of neuromuscular fatigue.

Although the PWC_FT_ has been used as a surrogate measure of neuromuscular fatigue, initial studies focused on identifying key factors that influence the PWC_FT_ [19]. For example, Housh and colleagues [20] examined how the PWC_FT_ was influenced by glycogen depletion and supercompensation. The authors reported no significant means differences between the PWC_FT_ values for the two experimental conditions. Similar Housh et al. [21] reported that the PWC_FT_ was not influenced by ammonium chloride and sodium bicarbonate ingestion. Other studies have found that supplementation with beta-alanine [22], arginine [23], or creatine [24,25] does improve PWC_FT_ and, therefore, delay the onset of neuromuscular fatigue. Recently, Plasencia et al. [4] reported that FM significantly increased PWC_FT,_ which in turn, delayed the onset of neuromuscular fatigue. One potential limitation of the study is that a single-joint exercise was used which may not fully translate to multiple-joint movements like walking or cycling [4].

The purpose of this study, therefore, was to examine the influence of FM on a multi-joint exercise such as cycle ergometry. We hypothesized that FM would delay on the onset of the PWC_FT_ relative to the no FM (i.e., control) condition. Secondarily, we hypothesized that FM would increase the mean maximal power output achieved during the incremental cycle ergometer test.

## 2. Methods

### 2.1. Overall Design and Implementation

Our inclusion criteria included no known cardiovascular, respiratory, and/or neuromuscular diagnosis. This information was provided by each subject using a detailed health history questionnaire. Following a screening visit to determine the eligibility of potential subjects, each subject visited the laboratory on two occasions each separated by 7 days. This 7-day wash-out period is typically used to eliminate muscle soreness associated with performing an incremental cycle ergometer test to voluntary exhaustion [4,26]. During the experimental condition (FM) visit, subjects performed a 5 min warm-up on a cycle ergometer unloaded (0 W), received 14 min of FM (7 min per leg), and then had EMG electrodes placed on the vastus lateralis muscle of non-dominant (based on kicking preference) limb [4,26]. Thereafter, each subject performed an incremental cycle ergometry test to voluntary exhaustion. For the visit where the subject received no FM (control condition), each subjects performed a 5 min warm-up on a cycle ergometer unloaded (0 W), rested for 14 min, had EMG electrodes placed on both vastus lateralis muscles, and then performed the incremental cycle ergometry test [4,26]. The order of the visits was randomized for each subject. The PWC_FT_ values for the two visits were estimated separately using the EMG data from each corresponding test.

### 2.2. Subjects

Based on an average effects size (Cohen’s *d* = 1.61) from previous studies [4,26,27] on PWC_FT_, an a priori power analysis indicated that a sample size of *N* = 8 was sufficient to detect a mean difference between the conditions at 90% statistical power. Thus, twelve healthy college-aged men ranging from 23 to 31 years (mean ± SEM: age, 25.0 ± 0.4 years; body mass, 86.4 ± 4.2 kg; and height, 1.80 ± 0.03 m) volunteered for this study. All subjects performed recreational activities such as basketball, weightlifting, and jogging, but none were collegiate athletes. All subjects were instructed to maintain their regular exercise and dietary routines throughout the study [4,26]. Twenty-four hours prior to their testing session, subjects were instructed to avoid lower body exercise [4,26]. Moreover, to minimize diurnal effects, all subjects visited the laboratory at the same time of day (±1 h) [4,26]. All subjects signed an approved Informed Consent by the University Institutional Review Board for Human Subjects.

### 2.3. Functional Massage (FM) Protocol

FM was performed on the vastus lateralis during experimental session. A licensed physical therapist, who is also a co-investigator, administered the FM. This therapist holds a graduate certificate in orthopedic manual physical therapy (OMPT), a clinical specialty requiring two years of advanced training beyond an entry-level physical therapy degree. OMPT certification focuses on advanced theoretical and clinical skills, emphasizing manual therapy techniques such as functional massage. Furthermore, the co-investigator has 26 years of clinical experience in outpatient orthopedics and sports medicine, where FM has been a consistent part of the management of lower extremity injuries.

The FM technique and positioning used in this study replicated common practices in physical therapy and sports medicine. Before the FM session, subjects were instructed to sit on a Cardon therapy table (Cardon Rehabilitation & Medical Equipment Ltd., Niagara Falls, NY, USA). The tibiofemoral joint line was positioned 2–3 inches distal to the table’s edge to allow the necessary passive knee flexion during the FM.

A 7 min lengthening active assisted FM technique to each limb. This involved compressing the vastus lateralis muscle toward the underlying bone with one hand while passively flexing the knee with the other hand to gently lengthen the muscle. The subject then actively assisted in returning the knee to full extension. These steps were repeated to allow the FM to be performed rhythmically and continuously, with the muscle compression progressing distally from the muscle origin at the anterior hip to the musculotendinous junction just superior to the patella.

### 2.4. Electrode Placement

Subjects shaved the area of the vastus lateralis where EMG electrodes were placed on both thighs. Bipolar EMG electrodes (EL500-6, BIOPAC Systems, Inc., Santa Barabara, CA, USA) with a 20 mm center-to-center interelectrode distance were used, consistent with our previous work [4,26]. The specific placement of the electrodes was determined using the recommendations of SENIAM (Surface ElectroMyoGraphy for the Non-Invasive Assessment of Muscles) [28]. Briefly, two-thirds of the distance from the Anterior Spina Iliaca Superior (ASIS) to the lateral side of the patella was measured in both thighs. In order to maintain electrode placement consistently for the subsequent visit, a permanent marker was used to outline the electrode location and subjects were instructed to trace over the line if it started to fade [4,26]. A third reference electrode was placed over the iliac crest [4,26]. Lastly, the EMG signal was amplified (gain: ×1000) using differential amplifiers (EMG 100B, BIOPAC Systems, Inc., Santa Barbara, CA, USA).

### 2.5. Incremental Cycle Ergometry

Each subject performed an incremental cycle ergometry test on a calibrated electronically braked cycle ergometer (Monark 829E; Country Technology, Inc., Gays Mills, WI, USA) at a pedaling cadence of 70 revolutions per minute [4,26]. In addition, seat height was adjusted so that the subject’s knees were slightly bent (~5° bend) during the downstroke phase of pedaling. This information was recorded for each subject for the subsequent visit [4,26]. Subjects started a 2 min warm-up at unloaded pedaling (0 W) and, thereafter, the power output increased 15 W per minute until voluntary exhaustion. The exercise test was terminated if subjects met at least two of the following three criteria: (1) 90% of age-predicted heart rate (220 − age); (2) Modified Borg rating of perceived exertion of ≥8 (scale of 0 to 10); (3) inability to maintain the pedal cadence of 70 revolutions per min despite strong verbal encouragement [4,26].

### 2.6. Determining the PWC_FT_

**Figure 1:**
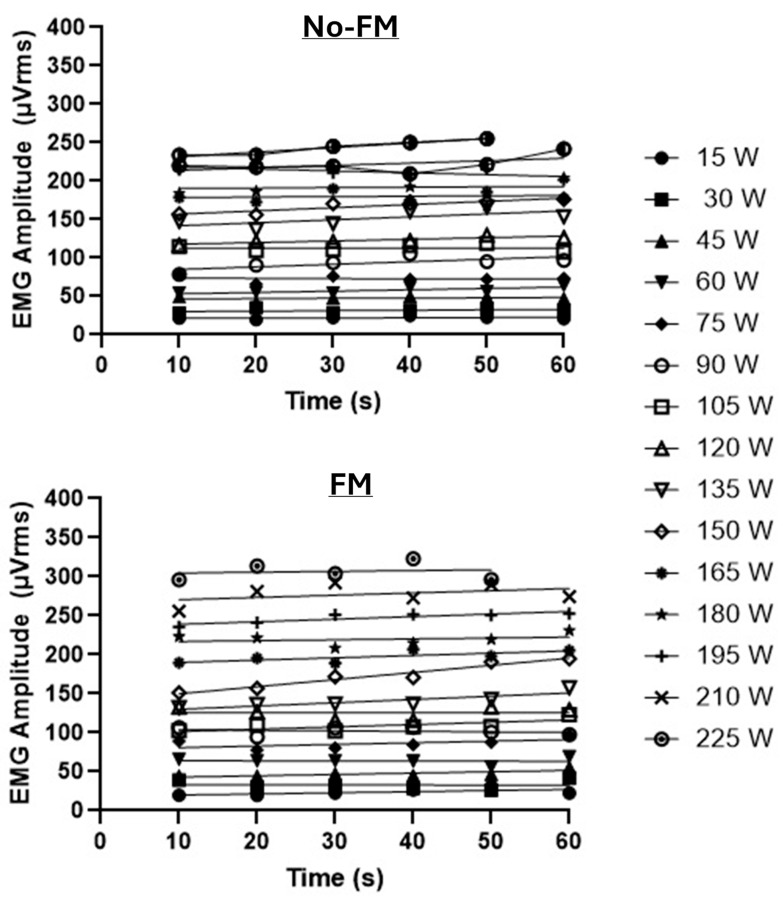
Depiction of the incremental cycle ergometer test for the no-FM (**top panel**) and FM conditions (**bottom panel**) in the same subject. For the no-FM condition, the PWC_FT_ (105 W + 120 W)/2 = 112.5 or 113 W, whereas for the FM condition, the PWC_FT_ (120 W + 135 W)/2 = 127.5 or 128 W.

For each subject across both visits, the PWC_FT_ was determined by calculating the absolute EMG amplitude (in microvolts root mean square, µVrms) using 10 s sampling windows at each power output level [4,26]. Separate linear regressions were performed for the EMG amplitude versus time relationship for each power output. The highest power output with a non-significant (*p >* 0.05) slope term was identified as well as the lowest power output with a significant (*p <* 0.05) slope term [4,26]. The average of these two power outputs was the PWC_FT_. As shown in Figure 1, each subject had a corresponding PWC_FT_ value for the no FM and FM test conditions [4,26].

### 2.7. EMG Signal Acquisition and Processing

The raw EMG signals were digitized at 1000 Hz and stored in a personal computer (Dell Inspiron E1705, Dell Inc., Round Rock, TX, USA) for subsequent analysis. All signal processing was performed using custom programs written with LabVIEW programming software (version 2019, National Instruments, Austin, TX, USA). The EMG signals were bandpass filtered (fourth-order Butterworth) at 10–500 Hz. The amplitude value for each stage was calculated for each subject based on the average of all the completed bursts during the sampling window [4,26].

### 2.8. Statistical Analysis

All data in Table 1 are presented as mean ± SEM (standard error of the mean) values. Mean differences between outcome variables such as maximal power output, absolute and normalized PWC_FT_, absolute and normalized heart rate at end-exercise, and RPE at end-exercise were analyzed with separate paired samples *t*-tests. All statistical analyses were performed using Statistical Package for the Social Sciences software (v. 28.0, IBM SPSS, Armonk, NY, USA) with an alpha level set at *p* ≤ 0.05.

## 3. Results

*Physiological Outcomes*

As shown in Table 1, the results of the separate paired-samples *t*-tests indicated significant mean differences for the absolute and relative PWC_FT_ mean values between the No-FM and FM conditions. For the remaining outcome variables, we observed no significant (*p >* 0.05) mean differences between the No-FM and FM for the remaining outcome variables.

## 4. Discussion

The principle finding of the present investigation was that FM performed on both legs prior to whole-body exercise resulted in an increase in PWC_FT_ relative to the control condition. These data support our hypothesis that FM delays the onset of neuromuscular fatigue. Conversely, the data did not support our hypothesis that FM would increase the mean maximal power output achieved from the incremental cycle ergometer test. The increase in PWC_FT_ during the FM visit is consistent with the findings of Plasencia and colleagues [4], who reported that FM increased the mean PWC_FT_ for incremental single-leg knee-extensor ergometer, relative to the control condition.

FM is a therapeutic technique employed in rehabilitation clinics by trained health professionals to enhance tissue extensibility, reduce pain, and improve overall function [1,2,3]. It involves engaging each specific muscle’s action through a combination of tissue lengthening and shortening while applying massage to the targeted area [1,2,3]. Typically performed for 5–12 min, FM is designed to improve range of motion and alleviate discomfort without inducing soreness or adverse effects [1,2,3]. Clinically, FM is often used when range of motion is limited due to muscular impairments or injuries [1,2,3]. Moreover, FM is commonly administered before exercise to improve range of motion without compromising muscle performance or after exercise to help relieve pain and muscle soreness [1,2,3]. Although used frequently in the clinic, empirical data validating FM remains limited, highlighting the need for further investigation to determine its efficacy.

The present study is a follow-up to the Plasencia et al. [4] study which examined the efficacy of FM on neuromuscular fatigue for the rectus femoris muscle during incremental single-leg knee-extensor ergometry using a within-subjects design. The investigators of the current study reported no statistically significant mean differences between the no-FM and FM conditions for maximal power output [4]. Plasencia and colleagues [4], however, reported a significant increase in PWC_FT_ (~80%) and PWC_FT_ as a percentage of maximal power output (%max, ~78%) for the FM condition relative to the no-FM condition. Moreover, there were no significant mean differences for other physiological outcomes such as end-exercise heart and end-exercise rate of perceived exertion for the exercised leg. In the current study, we found that PWC_FT_ and PWC_FT_ (%max) significantly increased by ~21% and ~18%, respectively (Table 1). Similarly to the findings of Plasencia and colleagues [4], we did not find significant mean differences between end-exercise heart rate and rate of perceived exertion indices between the two conditions. The magnitude of differences between the Plasencia et al. [4] and the current study for the PWC_FT_ index, however, resides in the mode of exercise. In the former study, the investigators used a single-joint, isolated muscle exercise (i.e., single-leg knee-extensor ergometry) [4], whereas in the latter study, we used a multi-joint, whole-body exercise (i.e., cycle ergometry). Previous studies have shown that during cycle ergometry subjects activate ~16% of the vastus lateralis muscle relative to their MVC (maximal voluntary contraction) [29]. Nevertheless, our findings, in conjunction with those of Plasencia et al. [4], indicate that FM performed prior to exercise can delay the onset of neuromuscular fatigue for incremental exercise paradigms.

The delayed onset in the neuromuscular fatigue, measured in the present study by PWC_FT_, may be potentially associated with postactivation potentiation (PAP). PAP refers to an increase in muscle force and rate of force development as a result of prior contractions [30,31]. For example, Franco and colleagues [32] concluded that different stretching paradigms decreased the time to reach peak power during a Wingate test compared to the no-stretching condition. The authors attributed these observed improvements, in part, to PAP [32]. Li and Liang [33] employed a unique set of four exercises (they collectively call the PAP protocol) to determine changes in a 250 m time trial in elite female cyclists. The investigators reported significant improvement in various cycle parameters using the PAP protocol relative to the control condition [33]. Typically, it has been suggested that PAP is most effective for athletes performing explosive, short-duration muscle action involving Type II muscle fibers [30,31] and the anaerobic energy system. Hamada et al. [34] investigated whether PAP was enhanced in endurance-trained athletes. The subjects (*N* = 40) were divided into four groups: triathletes, distance runners, active control subjects (recreational weight-trainers), and sedentary, untrained controls [34]. This study found that PAP, induced by a MVC, was enhanced in endurance athletes compared to active controls. Triathletes showed increased PAP in both upper and lower limb muscles, while distance runners exhibited enhanced PAP only in the lower limbs. These results suggest that PAP may help reduce fatigue during endurance activities [34]. Bremer and colleagues [35] reported that performing two incremental single-leg knee-extensor ergometry tests, with a 1 h rest between tests, resulted in a significant increase in PWC_FT_ (~27%) in the second test. The authors suggested that PAP may, in part, explain the improved performance for the second single-leg knee-extensor ergometer test [35]. Given that during FM the subject actively assisted in returning the knee to full extension for 7 min per limb, this may have been sufficient to induce PAP. Thus, the findings of Plasencia et al. [4] combined with the present study, suggest that PAP may be a contributing physiological mechanism underlying the delayed onset of neuromuscular fatigue observed during incremental exercise. The exact mechanism for the delay of fatigue is however unknown and other factors such as psychological preparedness, improved blood flow and oxygen delivery, metabolic buffering, improved recruitment of Type I fibers and reduced metabolic byproducts could contribute to the improved resistance to neuromuscular fatigue observed after FM.

Notwithstanding our findings, it is important to acknowledge a few limitations of the current study. First, we performed FM for 7 min per limb for a total of 14 min. In most rehabilitative settings, physical therapists may have a total of 30–45 min to work with their clients. Thus, dedicating a significant amount of the total time (up to ~31%) to FM may not be practical. Second, the subjects who volunteered for this study were healthy men without any musculoskeletal impairments. Thus, futures studies are needed to determine whether FM delays the onset of neuromuscular fatigue in different populations such as women and individuals with musculoskeletal impairments. Third, as suggested by Plasencia et al. [4], it would have significant practical implications to assess the efficacy of FM between events in sports where athletes compete in multiple events (e.g., swimming) within a single day. Thus, future studies are needed to determine whether FM improves performance following repeated exercise workloads.

In summary, the results of the current investigation showed that FM, performed prior to incremental cycle ergometry, improved PWC_FT_ by delaying the onset of neuromuscular fatigue. These findings are consistent with those reported by Plasencia and colleagues [4]. To our knowledge, objective measures of neuromuscular fatigue within a rehabilitative setting are limited. The PWC_FT_ index may provide a simple and practical methodology to assess the efficacy of a therapeutic intervention which is a departure from survey questionnaires which are subjective.

## Figures and Tables

**Table 1:** Results of the dependent variables (mean ± SEM).

	Experimental Conditions				
Outcome Variable	No-FM	FM	t-Statistic	*p-*Value	Cohen’s *d*
Maximal Power Output (W)	213 ± 13	218 ± 12	1.483	0.166	0.11
PWC_FT_ (W)	120 ± 8	145 ± 11	4.43	0.001	0.74
PWC_FT_ (%max)	57 ± 2	67 ± 3	3.21	0.008	1.04
End-exercise heart rate (bpm)	184 ± 3	182 ± 4	1.02	0.332	0.15
End-exercise heart rate (%max)	95 ± 2	93 ± 2	1.01	0.333	0.16
End-exercise RPE	10 ± 0	10 ± 0	1.15	0.275	0.36

Note: All subjects performed the No-FM and FM (functional massage) conditions in a randomized order; PWC_FT_ data are from the non-dominant limb; bpm: beats/min; %max: percent of maximal value for that condition; and RPE: rating of perceived exertion (0–10 scale).

## Data Availability

All data from the study are available from the authors upon reasonable request.
